# Association between dietary inflammatory index scores and the increased disease activity of rheumatoid arthritis: a cross-sectional study

**DOI:** 10.1186/s12937-022-00805-w

**Published:** 2022-08-16

**Authors:** Atiyeh Nayebi, Davood Soleimani, Shayan Mostafaei, Negin Elahi, Naseh Pahlavani, Amir Bagheri, Homayoun Elahi, Mahdi Mahmoudi, Seyyed Mostafa Nachvak

**Affiliations:** 1 Nutritional Sciences Department, School of Nutrition Sciences and Food Technology, Kermanshah University of Medical Sciences, Kermanshah, 6719851552 Iran; 2 Research Center of Oils and Fats, Kermanshah University of Medical Sciences, Kermanshah, Iran; 3 Division of Clinical Geriatrics, Department of Neurobiology, Care Sciences and Society, Karolinska Institute, Stockholm, Sweden; 4 Epidemiology and Biostatistics Unit, Rheumatology Research Center, Tehran University of Medical Sciences, Tehran, Iran; 5 Social Development and Health Promotion Research Center, Gonabad University of Medical Sciences, Gonabad, Iran; 6 Cellular and Molecular Research Center, Research Institute for Health Development, Kurdistan University of Medical Sciences, Sanandaj, Iran; 7 Department of Community Nutrition, School of Nutritional Sciences and Dietetics, Tehran University of Medical Sciences, Tehran, Iran; 8 Department of Rheumatology, Faculty of Medicine, Kermanshah University of Medical Sciences, Kermanshah, Iran; 9 Rheumatology Research Center, Tehran University of Medical Sciences, Tehran, Iran; 10 Inflammation Research Center, Tehran University of Medical Sciences, Tehran, Iran

**Keywords:** Diet, Dietary inflammatory index, Rheumatoid arthritis, Inflammation, Disease activity score

## Abstract

**Background:**

Diet plays an important role in regulating inflammation, which is a hallmark of rheumatoid arthritis (RA). Our aim was to investigate the association between the Dietary Inflammatory Index (DII) scores and RA activity.

**Methods:**

This cross-sectional study was conducted on 184 patients with RA in rheumatology clinic in Kermanshah city, Iran, in 2020. RA was diagnosed according to the criteria of the 2010 American College of Rheumatology/ European League against Rheumatism. The overall inflammatory potential of the diet was extracted from a validated 168-item food frequency questioner (FFQ) using the DII. RA disease activity was assessed using Disease Activity Score 28 (DAS-28) scores. Logistic regression and one-way ANOVA/ ANCOVA were conducted.

**Results:**

Individuals in the highest DII quartile had the significantly higher odds of positive C-reactive protein than those in the lowest quartile of the DII scores (OR 4.5, 95% CI 1.16 – 17.41, *P* = 0.029). A statistically significant downward linear trend in fat-free mass and weight were observed with increasing the DII quartiles (*P* = 0.003, *P* = 0.019, respectively). Patients in the highest DII quartile had higher DAS-28 scores than those in the first quartile (Mean difference: 1.16, 95% CI 0.51 – 1.81, *P* < 0.001) and second quartile of the DII scores (Mean difference: 1.0, 95% CI 0.34 – 1.65, *P* < 0.001).

**Conclusion:**

Our results indicated that reducing inflammation through diet might be one of the therapeutic strategies to control and reduce the disease activity in RA patients.

## Introduction

Rheumatoid arthritis (RA) is a systemic inflammatory autoimmune disease affecting about 1% of the general population at the prime of their lives. This disease is twice as common in women as in men, but the reason is not clear [1]. RA involves considerable inflammation, stiffness, swelling, pain, and destruction of joints that ultimately leads to erosive joint damage and progressive disability [2]. RA is associated with a higher risk of infection, chronic respiratory disease, cardiovascular disease, and cancer [3]. Furthermore, some medications prescribed in RA patients such as methotrexate and nonsteroidal anti-inflammatory drugs (NSAIDs) have adverse effects on renal function [4]. As RA is a multifactorial disease [5], the increased understanding of contributing factors to the progression or remission of disease can lead to the development of therapeutic approaches.

Patients with RA have significantly higher levels of C-reactive protein (CRP), interleukin (IL)-6, and tumor necrosis factor alpha (TNF-α) than healthy subjects [6]. Serum CRP levels in RA patients are strongly correlated with the disease activity, and the response to treatment usually does not occur unless the levels of this inflammation marker decrease [6, 7]. Diet is a modifiable environmental factor that plays an important role in regulating inflammation and immune function [8]. Some nutritional studies suggest that the intakes of foods with anti-inflammatory properties such as nuts, tea, fish, olive oil, and vegetables are associated with a lower risk or severity of RA [9, 10]. A recent clinical trial also shows that supplementation with fish oil can reduce disease activity score 28 (DAS-28) and the number of tender joints in patients with RA [11]. Nonetheless, the study of single foods is inadequate for taking into account cumulative effects among foods eaten together free-living people [12]. In this regard, a clinical trial shows that fish oil in combination with olive oil has more effects on improving RA [13]. In nutritional studies, several dietary quality indices have been designed to address this issue and assess the effects of overall diet.

In 2014, Shivappa et al. designed a literature-derived and population-based dietary index, named the dietary inflammatory index (DII), to quantify the overall inflammatory potential of diet [14]. DII has been shown in nutritional epidemiological studies to predict the relationship between overall diet and levels of inflammatory and anti-inflammatory markers including IL-1β, IL-4, IL-6, IL-10, CRP, and TNF-α [15, 16]. Recent evidence suggest using DII to predict the risk of diseases with an inflammatory etiology such as cardiovascular disease (CVDs) and mortality [17–19]. A recent case–control study revealed that inflammatory diets, indicated by higher DII scores, were associated with the increased odds of RA [20]. Although RA is a lifelong disease, further knowledge on the association between overall diet and RA can be helpful in the remission of the disease. In this study, we go one step further by evaluating whether higher DII scores indicate higher level of inflammatory diet.

## Materials and methods

### Study design and participants

This cross-sectional study was conducted on patients with RA attending the outpatient rheumatology clinic in Kermanshah city, Iran, in 2020. The protocol was approved by the ethics committee of Kermanshah University of Medical Sciences in accordance with the principle of the Helsinki Declaration (ID: IR.KUMS.REC.1400.219). All participants were simple randomly selected and they were informed of the objectives and benefits of the study as well as possible health risks at the time of enrollment. Then, written informed consent was obtained from each participant. Patients who were interested in participating were invited to a screening visit by the study rheumatologist using a simple random method. A key inclusion criterion was definite rheumatoid arthritis based on the criteria of the 2010 American College of Rheumatology/ European League against Rheumatism (ACR/EULAR) [21]. Briefly, the 2010 ACR/EULAR criteria for Rheumatoid arthritis include the number and location of involved joints (score from 0 to 5), serologic abnormality (score range 0–3), increased acute-phase response (score from 0 to 1), and symptom duration (score from 0 to 1). Participants who had at least a score of six with the confirmed presence of synovitis in at least one joint which is not better explained by another disease were diagnosed as definite RA. Other inclusion criteria were female gender (higher prevalence of RA, better cooperation, and consideration the participants’ homogeneity), an age of 18 years or older and written informed consent. Exclusion criteria were pregnancy, lactation, serious diseases (such as cancer, renal failure, and heart failure), other connective tissue or joint diseases (such as gout, lupus, and ankylosing spondylitis), inflammatory bowel disease, Cushing's syndrome, bone or joint surgery, and adherence to any specific dietary regimen. Finally, 184 eligible RA patients were included in this study based on the inclusion and exclusion criteria.

### Disease activity score 28

Disease activity score 28 (DAS-28) is a validated index for monitoring the disease activity of RA. This index is a multi-dimensional instrument that utilizes the number of swollen and tender joints, the levels of an acute phase reactant (CRP or ESR), and the total self-assessment of disease activity by visual analogue scale (VAS) of general health. In this study, all patients were examined by the study rheumatologist to determine the number of swollen joints (SJ) and tender joints (TJ), and then DAS-28 scores were calculated using the following formula [22]:

$$\mathrm{DAS}-28=\left[0.56\;\sqrt{\mathrm{TJ}}\right]+\left[0.28\;\sqrt{\mathrm{SJ}}\right]+\left[0.7\;\mathrm{Ln}\;\left(\mathrm{ESR}\right)\right]+\left[0.014\;\mathrm{VAS}\right]$$

### Dietary intake assessment

In the current study, a reliable and valid 168-item semi-quantitative food frequency questionnaire (FFQ) was used to assess usual dietary intake [23]. FFQs assess dietary intake during the last year ranking frequency of each food/beverage on a scale from “never or < 1 standard serving size per month” to “ ≥ 6 standard serving sizes per day”. Participants reported the daily, weekly, monthly, or annual consumption of each food item according to its standard serving size in the questionnaire. A trained dietitian collected dietary data from each participant through face-to-face interviews. The amount of each food item was converted to weight (grams/day) with the use of the Iranian household measures. Then, the energy and nutrients of each food item were obtained using the U.S. Department of Agriculture (USDA, release 22, 2009) food composition data subsumed in the Nutritionist IV software (First Databank Inc., Hearst Corp., San Bruno, CA, USA). We also used the Iranian food composition table for Iranian food items not enclosed in Nutritionist IV software [24]. Participants with a daily energy intake outside of predefined limits (± 3 standard deviation: 800–4200 kcal/day) were excluded from the final analysis [25–27].

### Dietary inflammatory index

DII is a literature-derived and population-based dietary index designated to quantify the overall effects of diet on inflammatory mediators. The scoring algorithm of DII includes 45 food parameters in which each parameter has an inflammatory effect score in the range of “-1” (maximum anti-inflammatory) to “ + 1” (maximum pro-inflammatory) based on its effect on six known markers (IL-1β, IL-4, IL-6, IL-10, CRP and TNFα) [28]. However, 32 food items (including: energy, carbohydrate, fiber, protein, total fat, monounsaturated fatty acid (MUFA), polyunsaturated fatty acid (PUFA), saturated fatty acid (SFA), trans fat, *n*-3 fatty acids, *n*-6 fatty acids, cholesterol, vitamin B1, vitamin B2, vitamin B3, vitamin B6, vitamin B9, vitamin B12, vitamin C, vitamin A, vitamin D, vitamin E, β-Carotene, zinc, selenium (Se), magnesium (Mg), iron (Fe), caffeine, onion, garlic, pepper, and green/black tea) were available for calculating the DII. In the DII calculation process, we subtracted the intake of each food parameter from the relevant global mean intake and then divided it by the global standard deviation intake to obtain a Z score. This Z score is converted to percentile score, then doubled and subtracted from “1”. This value is multiplied by the corresponding inflammatory effect score to obtain the “food parameter-specific DII score”. Finally, all of the food parameter-specific DII scores are added to obtain a total DII score for each individual [28].

### Anthropometric assessment

A trained dietitian assessed the weight and height of all participants under the standard conditions. Weight was measured with minimal clothing and without shoes to the nearest 0.1 kgusing a digital scale (Seca 831, Hamburg, Germany). Height was measured without shoes to the nearest 0.1 cmusing a wall-mounted stadiometer (Seca 206, Hamburg, Germany). Then, body mass index (BMI) was calculated by dividing weight in kilograms by the square of height in meters. Body fat mass (FM) and body fat-free mass (FFM) was measured with the bioelectrical impedance analysis (BIA) method using a Tanita BC- 418 body composition analyzer (Tanita Corp., Tokyo, Japan). Before the BIA measure, participants were asked to refrain from consuming caffeine-containing products and exercising and to be well hydrated [29].

### Biochemical assessment

Blood samples from all patients were obtained at the beginning of the study. Erythrocyte sedimentation rate (ESR) that determination is an essential base for patient disposition with rheumatologic conditions and a surrogate marker of the acute phase reaction and is affected by rising concentrations of fibrinogen, the main clotting protein, and alpha globulins during an inflammatory reaction was measured using the Westergren technique [30]. Ethylene-diamine tetra-acetate (EDTA) potassium anticoagulant was added to the samples and diluted with 0.85% NaCl solution and poured into Westergren pipe with ant “0” (1 mm) sample. The distance between the top of the deposited erythrocyte column and the plasma meniscus was recorded within 1 h/mm. The samples also were centrifuged at 3000 rpm for 15 min for measuring the titration of anti-nuclear antibodies (ANA) and antibodies against cyclic citrulline peptide (anti-CCP) using the ELISA method (Seramun Diagnostica GmbH, Heidesee, Germany) and rheumatoid factor (RF) and C-reactive protein (CRP) using the latex agglutination turbidimetric immunoassay. ANA is a various group of autoantibodies that are important to the assessment of patients with a wide range of rheumatic diseases and usual properties of autoimmune connective tissue diseases. These antibodies are helpful biomarkers for screening and diagnosis and provide insights for comprehending disease mechanisms [31, 32]. Anti-CCP is among the most specific autoantibody systems explained in RA and has a role in the pathogenesis of the disease [33]. Also, it is very specific for RA and is appeared in at least 60–75% of the patients studied. so, it is a new and important serologic diagnostic marker for the diagnosis of RA [34]. RF is an autoantibody that is directed to immunoglobulin G and the most characteristic of the autoimmune response is the presence of such antibodies [35]. RF find in up to 75% of patients with RA depending on the stage of the disease. The value of these antibodies is usually related to the diagnosis and prognosis of diseases [36]. CRP is a nonspecific acute-phase reactant that is an integral part of the innate immune system. It is synthesized in response to inflammation and/or tissue injury, and its increment is proportionate with inflammatory mediators (cytokines) produced by cells actively participating in the milieu of tissue injuries such as IL-1, IL-6, TNF-α and TGF-β. The level of CRP tends to be proportional to the severity of the inflammatory process. So, levels of this marker are sensitive to narrow changes in the acute-phase response [37].

### Statistical analysis

All statistical analyses were performed using SPSS 16 software. Kolmogorov–Smirnov test was used to determine the normality distribution of quantitative variables. Quantitative variables with normal distribution are presented as mean ± standard deviation (SD), quantitative variables with non-normal distribution are presented as median [interquartile range], and others are presented as numbers (percentage). One-way ANOVA with Tukey’s Post-hoc test was used to analyze quantitative variables with normal distribution, and Kruskal–Wallis H test was used to analyze quantitative variables with non-normal distribution. The odds ratio and its 95% CI for assessing the associations between qualitative variables (e.g. CRP) and DII quartiles was calculated by the binary logistic regression. Adjusted means of DAS-28 scores across the DII quartiles were obtained using ANCOVA. *P*-value of less than 0.05 was considered as statistically significant.

## Results

The current study consisted of 184 eligible patients with RA. All patients received medication for the treatment of rheumatoid arthritis, including Corticosteroids, Disease modifying antirheumatic drugs (DMARDs) such as Methotrexate, Hydroxychloroquine, Sulfasalazine, Azathioprine, and biological DMARDs. The mean age was 49.1 years (SD = 12.91), mean weight was 69.21 kg (SD = 13.34) the mean BMI was 27.25 kg/m^2^ (SD = 5.11). The most of the patients were a housekeeper (90.8%) and had a positive CRP (85.3%).

The general characteristics of the RA patients according to the quartiles (Q) of the DII scores are shown in Table 1. The mean age and median duration of disease did not differ significantly among the quartiles of DII scores (*P* > 0.05). In addition, the distribution of the patients in the terms of having a positive ANA, a positive anti-CCP, a positive RF, and university education and using dietary supplements was not significantly different across the quartiles of the DII scores.

**Table 1:** General Characteristics of participants across quartiles (Q) of the Dietary Inflammatory Index (DII) scores

Variables	Quartiles of the Dietary Inflammatory Index scores				*P*-value
	Q1 Lower Inflammatory Diet	Q2	Q3	Q4 Higher Inflammatory Diet	
DII, median	- 2.31	- 0.99	- 0.48	+ 0.68	-
Number	46	46	46	46	-
Age; years	46.67 ± 13.37	48.63 ± 10.10	50.04 ± 14.52	51.08 ± 13.24	0.618†
Positive ANA; n (%)	27 (58.7%)	29 (63%)	34 (73.9%)	33 (71.7%)	0.108‡
Positive anti-CCP; n (%)	42 (91.3%)	43 (93.5%)	43 (93.5%)	45 (97.8%)	0.607‡
Positive RF; n (%)	42 (91.3%)	43 (93.5%)	44 (95.7%)	43 (93.5%)	0.870‡
Use of Supplement; n (%)	39 (84.8%)	35 (76.1%)	34 (73.9%)	29 (63%)	0.112‡
University education, n (%)	7 (15.2%)	8 (17.4%)	5 (10.9%)	10 (21.7%)	0.558‡
Duration of disease; years	15 [6–28]	11 [4–22]	16 [6- 30.5]	20 [9 – 26.5]	0.243*

Data are presented as a mean ± standard deviation, median [interquartile range], or frequency (percentage)

*Abbreviations. DII* Dietary inflammatory index, *ANA* Anti-nuclear antibodies, *CCP* Cyclic citrulline peptide, *RF* Rheumatoid factor, *CRP* C-reactive protein

^†^ *P*-value was obtained from the one-way ANOVA test

^‡^ *P*-values were obtained from the chi-squared test

^*^P-value was calculated using the Kruskal–Wallis test

The values of the anthropometric characteristics and energy intake of participants according to the quartiles of the DII scores are shown in Table 2. The mean energy intake, weight, fat-free mass, and fat mass showed a significant difference between the quartiles of DII scores (*P* < 0.05). The further analysis showed a linear trend toward decreasing fat-free mass, weight, and energy intake in patients with increasing quartiles of the DII scores (*P*-trend = 0.003, *P*-trend = 0.019 and *P*-trend = 0.014 respectively). The fat-free mass trend remained significant after adjusting the effect of weight (*P*-trend = 0.040). The mean BMI and waist circumference were not significantly different across the quartiles of DII scores (*P* > 0.05).

**Table 2:** Anthropometric characteristics and energy intake of participants across quartiles (Q) of the Dietary Inflammatory Index (DII) scores

	Quartiles of the Dietary Inflammatory Index scores				
Variables	Q1 Lower Inflammatory Diet	Q2	Q3	Q4 Higher Inflammatory Diet	*P*-value†
DII, median	- 2.31	- 0.99	- 0.48	+ 0.68	-
Number	46	46	46	46	-
Energy intake, Kcal/day	3543 ± 370	3726 ± 367	3451 ± 576	3074 ± 597	0.001
Weight; kg	70.61 ± 15.21	70.79 ± 12.06	71.93 ± 11.97	63.48 ± 12.57	0.008
Body mass index; kg/m^2^	27.19 ± 5.34	27.71 ± 5.09	28.41 ± 4.80	25.67 ± 4.92	0.066
Fat mass; kg	24.51 ± 9.49	25.38 ± 8.02	26.23 ± 7.07	21.31 ± 7.92	0.026
Fat free mass; kg	46.10 ± 6.64	45.41 ± 5.24	45.71 ± 5.61	42.16 ± 5.43	0.004
Waist circumference; cm	93.52 ± 11.47	93.51 ± 15.76	96.52 ± 9.37	92.00 ± 9.48	0.318

Data are presented as a mean ± standard deviation

*Abbreviation. DII* Dietary inflammatory index

^†^ *P*-value was obtained from the one-way ANOVA test

**Fig. 1:**
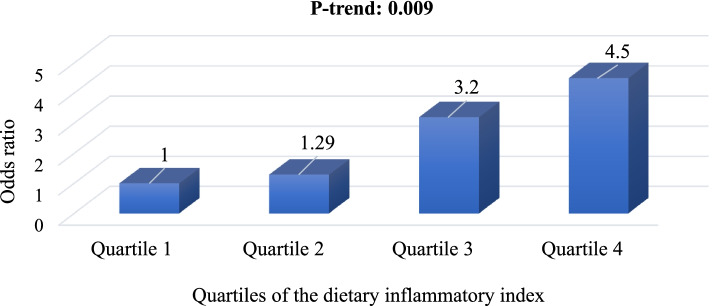
Odds ratio of having a positive C-reactive protein across the quartiles of the dietary inflammatory index. *P*-trend was obtained from the Binary Logistic Regression

**Fig. 2:**
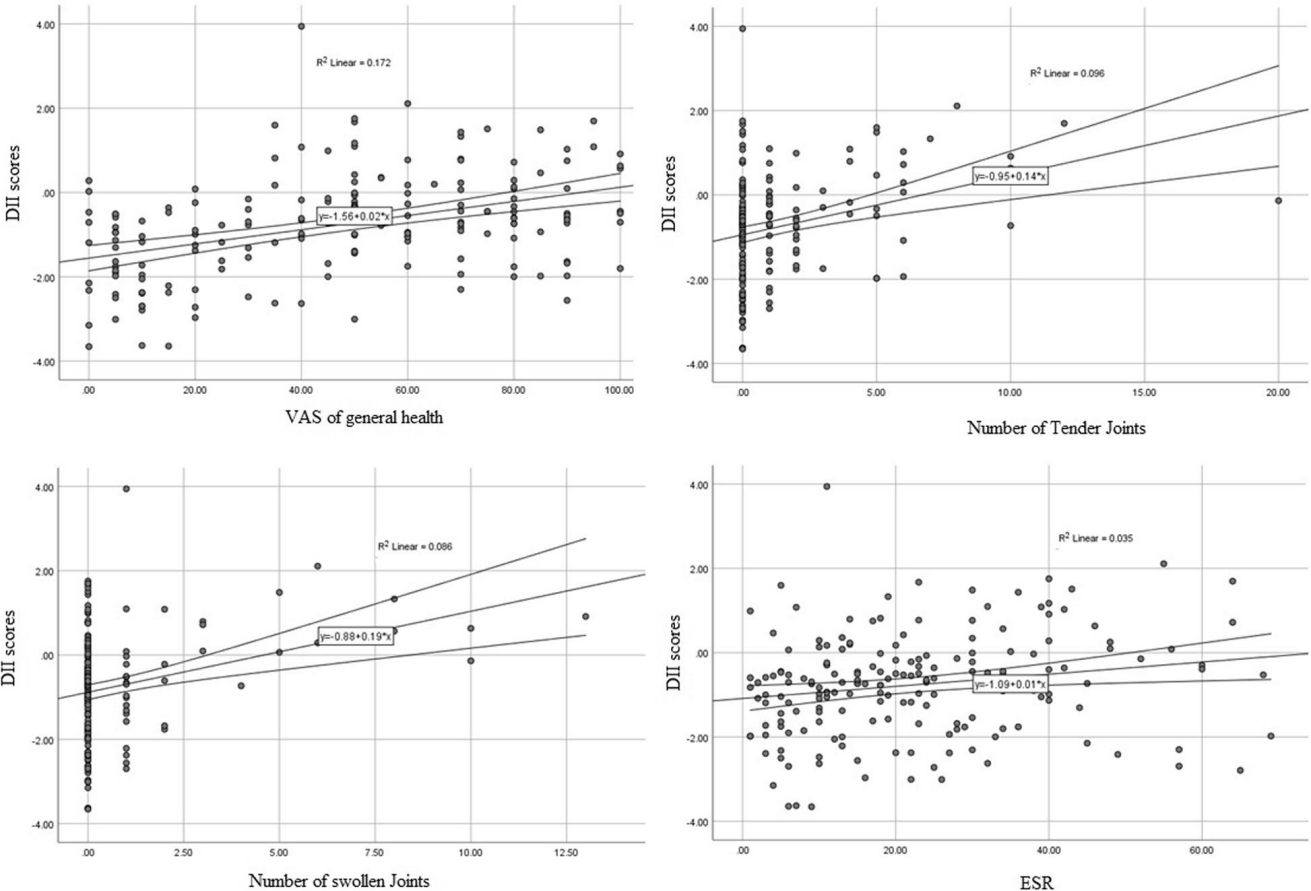
Relationship of dietary inflammatory index with individual factors of DAS-28 scores

The odds of having a positive CRP across the quartiles of the DII scores are illustrated in Fig. 1. The patients in the highest DII quartile (higher inflammatory diet) had the higher odds of having a positive CRP than those in the lowest quartile of the DII scores (OR 4.5, 95% CI 1.16 – 17.4, *P* = 0.029). Totally, a significant upward linear trend in the odds of having a positive CRP was observed across the quartiles of the DII scores (*P*-trend = 0.009). The mean of DAS 28 scores of patients with RA across the quartiles of the DII scores are illustrated in Table 3. In the crude model, the patients in the highest DII quartile had the higher DAS 28 scores than those in the lowest quartile of the DII scores (Mean difference: 1.16, 95% CI 0.51 – 1.81, *P* < 0.001). In the energy-adjusted model as the adjusted model 1, the patients in the highest DII quartile had the higher DAS 28 scores than those in the lowest quartile of the DII scores (Mean difference: 1.51, 95% CI 0.99 – 2.09, *P* < 0.001). In the adjusted model 2, after the adjustment of potential confounding factors including energy intake, age, fat mass, fat free mass, weight, duration of disease, and supplement use, the patients in the highest DII quartile had the higher DAS 28 scores than those in the lowest quartile of the DII scores (Mean difference: 1.68, 95% CI 1.02 – 2.13, *P* < 0.001). Totally, significant upward linear trends were observed in the mean of DAS 28 scores across the quartiles of the DII scores (*P*-trends < 0.001) in all of the models (Table 3). Figure 2 shows the association of dietary inflammatory index with individual factor which were included in DAS-28 score. There was a significant relationship of DII scores with VAS of general health (correlation coefficient: 0.41, *P* = 0.001), number of swollen joints (correlation coefficient: 0.29, *P* = 0.001), number of tender joints (correlation coefficient: 0.31, *P* = 0.001), and ESR (correlation coefficient: 0.19, *P* = 0.01).

**Table 3:** The mean of DAS-28 scores across the quartiles of dietary inflammatory index

Model	**Quartiles of the Dietary Inflammatory Index scores**				*P*-Trend†
	Q1 **Lower Inflammatory Diet**	Q2	Q3	Q4 **Higher Inflammatory Diet**	
Crude Model	2.58 (2.24 – 2.93)	2.75 (2.41 – 3.52)	3.18 (2.84 – 3.52)	3.74 (3.14 – 4.09)	< 0.001
Adjusted Model 1	2.44 (1.84 – 2.63)	2.68 (2.19 – 2.92)	3.18 (2.79 – 3.50)	3.95 (3.53 – 4.23)	< 0.001
Adjusted Model 2	2.23 (1.86 – 2.65)	2.46 (2.22 – 2.95)	3.23 (2.79 – 3.51)	3.91 (3.47 – 4.19)	< 0.001

Data are presented as a mean (95% CI)

*Abbreviation.* DII: dietary inflammatory index

Model 1: Adjusted for energy intake

Model 2: Adjusted for energy intake, age, fat mass, fat free mass, duration of disease, weight, and supplement use

^†^*P*-trends were obtained from the analysis of covariance

## Discussion

The main finding of the current study was an association of the higher inflammatory diet with the increased activity of rheumatoid arthritis disease, independent of potential confounding factors. This study also provided additional evidence of the relationship of the dietary inflammatory index with inflammatory markers such as CRP and ESR. A significant upward linear trend in the odds of having a positive CRP was observed across DII quartiles scores. Moreover, there was a significant relationship of DII scores with fat free mass, VAS of general health, number of swollen joints, number of tender joints and ESR.

The finding indicated that the higher inflammatory potential of diet was associated with the elevated activity of rheumatoid arthritis disease and its related factors including ESR, VAS of general health, number of swollen joints, number of tender joints and CRP. To our knowledge, there was limited study conducted on the association of DII score and activity of rheumatoid arthritis disease and its components. Similar to our findings, Tandorost et al. in a study on patients with rheumatoid arthritis reported that the highest DII had significantly higher serum inflammatory (hs-CRP and TNF) and clinical markers (The number of tender joints and DAS-28 score) [38]. In a cohort study by Dainty et al. in the UK Biobank population was shown a significant association between inflammation (CRP) and diet and RA [39]. Also, diets having high amounts of fruits and vegetables are associated with low levels of hs-CRP [40]. In a cross-sectional and longitudinal study conducted in Japan showed that anti-inflammatory change in DII score was related with low disease activity [41]. According to the findings of a case–control study, adherence to an inflammatory diet such as Western Diet increases the chances of developing RA, while following a healthy diet can reduce the incidence of the disease [42]. In fact, unhealthy dietary patterns like high DII diets due to their high glycemic load and the presence of certain components such as refined carbohydrates and sweeteners in them can cause harmful effects such as overproduction of free radicals and reduced total antioxidant capacity (TAC), insulin resistance, and disruption of lipid profile levels [43–45]. These conditions ultimately lead to non-communicable chronic diseases (NCCD) such as diabetes, obesity, and RA. Previous observational studies have shown that consuming rich sources of omega-3 fatty acids such as fish (twice a week) can be associated with DAS-28 reduction [46]. A meta-analysis of 20 published randomized controlled trials (RCTs) has shown that omega-3 fatty acids supplementation reduces eight disease-activity–related markers in patients with RA [47]. According to recent recommendations, RA patients should restrict red meats, processed meats, sugar, and beverages on diet, substitute green tea for coffee, and increase the intake of antioxidants, phytochemicals, vitamins, and flavonoids to reduce inflammation and complications of the RA disease [48]. Results of a rheumatoid arthritis registry indicated that sugar and desserts might aggravate symptoms in arthritis patients, while spinach, fish, and blueberries can improve them [49]. In another study by Linos et al., it was shown that high consumption of olive oil with cooked vegetables in the diet reduces the risk of arthritis, which is probably due to the immune-boosting effects of olive oil as well as high levels of phenolic compounds and antioxidants in vegetables that reduce the levels of inflammatory mediators in the body [50, 51]. Therefore, it seems that adherence to a diet with low DII scores might be helpful in reducing the symptoms and disease activity in patients with RA.

Another result of the study showed that the amount of fat-free mass decreases with increasing the inflammatory index of the diet. In line with this result, previous studies showed that with the increase of inflammatory markers in the body, the amount of fat-free mass decreases [52, 53]. Inflammation causes more muscle loss by increasing muscle catabolism, which in turn can lead to weight loss in the advanced stages of arthritis [54, 55]. Evidence suggests that cytokines such as TNF-α and IL-1β are elevated in patients with RA [56]. Increased cytokines lead to chronic systemic inflammation in these patients. This pattern of inflammation in patients with RA increases the chances of developing certain diseases such as cancer [57] and atherosclerosis [58]. Reducing pro-inflammatory cytokines and consequently reducing inflammation is one of the important approaches in the treatment of RA. This is while drug treatments to reduce inflammation in patients with RA are usually associated with some side effects [58]. Increase in pro-inflammatory cytokines is one of the effects of inflammatory diets in the body [59]. Therefore, there is a strong possibility that consuming diets with high inflammatory index by patients with RA can lead to worsening of the disease and its symptoms. Conversely, by consuming an anti-inflammatory diet, there is a possibility of reducing the severity of the disease and pain in patients with RA due to the reduction of pro inflammatory factors. Some studies have shown the effect of anti-inflammatory diets on reducing the complications of arthritis [60]. Anti-inflammatory diets can be considered as a valuable and effective approach to treatment of RA as they have no side effects and are economical, comfortable and compatible with life. We suggest that in the protocols and guidelines related to the treatment of RA, dietary assessment of patients be considered as a part of treatment and according to this assessment the necessary recommendations to follow an anti-inflammatory diet should be educated and monitored.

Our study was strengthened by adjustment for potential confounding factors and the similarity among DII quartiles in terms of general characteristics. The limitation of the present study is the fact that it cannot conclude a cause–effect relationship between inflammatory potential of diets and activity of RA because of the cross-sectional design. Moreover, this study was conducted only among women, and the results may not be generalizable to men.

## Conclusion

This study concludes that patients with rheumatoid arthritis often follow a poor diet with a high inflammatory index, so our results show that adherence to a diet with a low DII can lead to decreasing the level of inflammatory factors and reduce the DAS28 levels that cause the decrease of disease activity in these patients. Prospective longitudinal studies or clinical trials are needed to elucidate whether the low DII diet can induce relevant improvements in RA disease activity.

## Data Availability

The data that support the findings of this study are available on request from the corresponding author.

## Abbreviations

RA: Rheumatoid Arthritis
FFQ: Food Frequency Questioner
DII: Dietary Inflammatory Index
DAS28: Disease Activity Score 28
CRP: C-reactive protein
NSAIDs: Nonsteroidal anti-inflammatory drugs
ACR/EULAR: American College of Rheumatology/ European League against Rheumatism
VAS: Visual Analogue Scale
ESR: Erythrocyte Sedimentation Rate
SJ: Swollen Joints
TJ: Tender Joints
MUFA: Monounsaturated fatty acid
PUFA: Polyunsaturated fatty acid
SFA: Saturated fatty acid
BMI: Body Mass Index
FM: Fat Mass
FFM: Fat-free mass
BIA: Bioelectrical impedance analysis
ANA: Anti-nuclear antibodies
Anti-CCP: Antibodies against cyclic Citrulline Peptide
RF: Rheumatoid Factor
TAC: Total Antioxidant Capacity
NCCD: Non-communicable chronic diseases
DMARDs: Disease Modifying Antirheumatic Drugs
